# Programme-level redesign and student outcomes in specialist post-registration ophthalmic nursing education: a cohort study

**DOI:** 10.1186/s12909-026-10029-9

**Published:** 2026-07-25

**Authors:** Inês Martins Esteves

**Affiliations:** 1 Department of Public Health and Caring Sciences, Uppsala University, BMC, Husargatan 3, Box 564, Uppsala, 751 22 Sweden; 2 School of Medicine and Biomedical Sciences (ICBAS), University of Porto, Porto, Portugal; 3 Health Sciences Research Unit: Nursing (UICISA:E), Nursing School, University of Coimbra, Coimbra, Portugal; 4 RISE-Health, Nursing School, University of Porto, Porto, Portugal

**Keywords:** Health professions education, Nursing education, Ophthalmic nursing, Post-registration education, Curriculum design, Assessment, Programmatic assessment, Competency-based education, Educational outcomes

## Abstract

**Background:**

Post-registration specialist nursing programmes must balance academic standards with workforce responsiveness, but fragmentation often persists across learning outcomes, assessment structures, and workload distribution. Although theoretical models including constructive alignment, programmatic assessment, competency-based education, and cognitive load theory promote coordinated design, empirical evaluation of integrated programme-level reforms remains limited, particularly regarding structural change and its association with measurable educational outcomes.

**Methods:**

This retrospective observational cohort study examined the association between a theory-informed programme-level structural redesign and student academic achievement in a United Kingdom (UK) post-registration specialist ophthalmic nursing programme. The study also introduces the Integrated Programme Reform Model (IPRM) as a framework for programme-level educational reform. Nine sequential cohorts of registered nurses were included (*N* = 106). Six cohorts completed the traditional programme structure (2018–2021; *n* = 73), and three cohorts completed the redesigned programme (2022–2023; *n* = 33). The redesign integrated strategies derived from alignment, programmatic assessment, competency-based progression, and workload redistribution. First-attempt summative assessment outcomes were compared using Welch’s t-tests, Mann-Whitney U tests, and Fisher’s exact tests. Effect sizes were calculated using Cohen’s d.

**Results:**

Following programme redesign, mean student achievement increased from 57.4% (SD 10.6) to 71.1% (SD 8.1), with a mean difference of 13.7% points (95% CI 10.0–17.4; *p* < 0.001; Cohen’s d = 1.39). The proportion of students achieving distinction (≥ 70%) rose from 15.1% to 60.6% (*p* < 0.001), while the proportion of scores below the standardised 50% pass threshold fell from 19.2% to 0% (*p* = 0.005). These findings indicate a distribution-wide upward shift in achievement across the full performance spectrum, alongside reduced variability following redesign.

**Conclusions:**

Coordinated programme-level structural redesign was associated with sustained higher first-attempt academic achievement across successive cohorts. Causality cannot be inferred: the redesign coincided with a transition to master’s-level provision and a change in assessment modality, the evaluation was conducted within a single programme by an author professionally involved in the redesign, and the data are historical. The IPRM is offered as a conceptual design heuristic for organising programme-level reform and requires prospective, independent, multi-site evaluation before any claim of effectiveness or transferability can be made.

## Introduction

A specialist programme may appear coherent on paper, with each module and assessment individually defensible, yet still accumulate structural misalignment as it evolves. Whether correcting that misalignment changes what students achieve is rarely examined directly. Post-registration specialist nursing programmes must develop advanced clinical and professional capabilities while remaining feasible for nurses engaged in demanding clinical roles. As specialist practice expands in autonomy and complexity, the internal structure of educational programmes becomes increasingly important. In this context, how learning, assessment, and competency processes are organised may be as influential as curriculum content. The present study is set within one such field: specialist ophthalmic nursing, a technically demanding post-registration domain in which registered nurses develop advanced capability for autonomous ophthalmic practice. Ophthalmic nurses increasingly work across technically complex assessment, triage, treatment-support, and patient-education roles, so curriculum architecture in this field must support both advanced clinical reasoning and a clear professional nursing identity.

These challenges are not unique to nursing and reflect wider concerns across health professions education regarding the relationships among curriculum architecture, assessment design, and learner outcomes. The problem is well recognised across the field: fragmentation between intended learning outcomes, teaching activities, assessment structures, and competency verification has been widely described across health professions education [1, 2]. When programmes evolve incrementally rather than through coordinated redesign, misalignment may accumulate over time, leading to curriculum drift, disproportionate workloads, and unclear progression pathways [3]. Research on curriculum mapping and structured levelling further suggests that, without deliberate sequencing and integration, competency development may progress unevenly across training stages [4]. In post-registration contexts, where learners balance academic study with ongoing clinical responsibilities, such fragmentation may reinforce surface learning strategies and create excessive assessment burden, particularly when extended written assignments dominate summative evaluation [5, 6].

Several theoretical traditions offer mechanisms to address these structural tensions. Constructive alignment emphasises explicit correspondence between intended outcomes, learning activities, and assessment tasks [7]. Programmatic assessment advances longitudinal, triangulated approaches to competency judgement across multiple data points [8]. Competency-based education foregrounds progression grounded in demonstrated professional capability rather than credit accumulation alone [9, 10], with recent scholarship highlighting the importance of coherent performance standards and integrated curriculum-assessment architectures in operationalising competency-based education effectively [11, 12]. From a learning-science perspective, cognitive load theory identifies the consequences of poorly structured assessment demands and excessive workloads [13]. Taken together, evidence from integrated and multi-component redesigns suggests that cohesive structural reform may strengthen alignment and improve learner outcomes when implemented systematically rather than incrementally [14, 15].

Despite these frameworks, few studies have rigorously evaluated coordinated, multi-component programme reform within United Kingdom (UK) post-registration nursing education over sustained timeframes. Existing research predominantly examines discrete interventions, which may be insufficient to shift outcomes when structural fragmentation is systemic. Longitudinal evidence on the effects of programme-level architectural reform therefore remains scarce [16].

This cohort-comparison study evaluates the transition from a historically evolved programme structure to a coordinated, theory-informed model of structural coherence within a UK specialist nursing programme. Drawing on six years of cohort data, this study examined the association between this transition and student academic achievement. Beyond reporting a local programme evaluation, this study contributes empirical evidence to the health professions education literature on programme-level curriculum and assessment redesign and introduces the Integrated Programme Reform Model (IPRM) as a framework for coherent, sustainable reform.

### Theoretical framework

The redesign evaluated in this study was conceived not as a series of isolated pedagogical modifications, but as a coordinated architectural reform grounded in complementary theoretical traditions that address programme-level fragmentation and assessment incoherence. Building on the fragmentation outlined above, the theoretical framework adopted here treats programme-level misalignment as a structural problem requiring coordinated, rather than component-level, response. In post-registration specialist contexts, fragmentation is amplified by parallel academic and clinical processes, high assessment volume, and limited integration of formative and summative requirements [5, 17].

Constructive alignment provides a foundational mechanism for restoring coherence by positioning intended learning outcomes as the organising principle of curriculum design [7]. Explicit alignment between outcomes, learning activities, and assessment tasks shifts attention from isolated innovations to structural relationships across the programme. Within postgraduate and specialist curricula, alignment has been associated with clearer expectations and more transparent progression [18, 19]. However, alignment alone does not define how competence should be conceptualised or how evidence should be aggregated longitudinally.

Competency-based education (CBE) extends this structural logic by reframing progression around demonstrable professional capability rather than time-based advancement [9, 10]. Effective CBE implementation requires coherent curriculum architecture, explicit competency mapping, and alignment between outcomes and assessment systems [12, 20]. In fragmented programmes, dense assessment structures may obscure developmental trajectories. CBE clarifies progression by anchoring curriculum design in authentic practice requirements.

Programmatic assessment provides the assessment architecture required to operationalise both alignment and CBE at the programme level. Rather than viewing assessment as a series of discrete events, programmatic assessment conceptualises it as an integrated system in which multiple data points are combined to inform defensible decisions about competence [8, 21, 22]. It emphasises proportionality, triangulation, and structured feedback processes, addressing the proliferation of isolated high-stakes tasks that may increase workload without strengthening validity.

Cognitive load theory (CLT) offers an additional rationale for structural reform [13]. Fragmented curricula may generate excessive extraneous cognitive load through duplicated content, task switching, and concentrated assessment demands. Evidence from integrated and concept-based redesigns suggests that deliberate sequencing and workload redistribution can strengthen curriculum coherence and support more consistent progression in learning [14]. From a cognitive load perspective, poorly structured learning environments may impose unnecessary load, which has been associated with reduced decision-making performance in workplace-based learning among nursing students [23]. For post-registration learners balancing employment and study, architectural decisions about workload distribution and assessment design are therefore educationally consequential rather than purely administrative [5, 6].

Taken together, these traditions form an integrated logic of structural reform: constructive alignment provides the organising blueprint, competency-based education defines developmental capability, programmatic assessment operationalises the integration of longitudinal evidence, and CLT clarifies the learning consequences of structural design. The Integrated Programme Reform Model (IPRM) synthesises these perspectives and conceptualises fragmentation as a system-level misalignment requiring coordinated architectural intervention; the present study evaluates this multi-component redesign as an applied example within post-registration specialist nursing education.

**Fig. 1:**
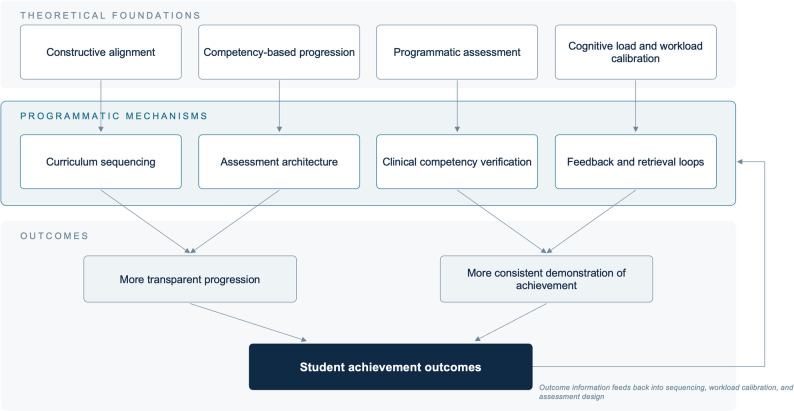
Integrated Programme Reform Model (IPRM). The model conceptualises programme-level redesign as the coordination of four interdependent design logics (constructive alignment, competency-based progression, programmatic assessment, and cognitive load and workload calibration), enacted through curriculum sequencing, assessment architecture, clinical competency verification, and feedback and retrieval loops. The model proposes that coherence across these mechanisms may support more transparent progression and more consistent demonstration of achievement, while recognising that empirical evaluation is required to establish transferability

Within the IPRM, these traditions are not parallel justifications but interacting components of a single design system. Constructive alignment supplies the organising spine linking intended outcomes, learning activities, and assessment; competency-based progression defines the developmental capabilities that this spine is aligned towards; programmatic assessment provides the architecture through which evidence of those capabilities is sequenced, triangulated, and aggregated into defensible judgements; and cognitive load and workload calibration condition whether that architecture is feasible for post-registration learners in clinical employment. Curriculum sequencing, assessment architecture, clinical competency verification, and feedback and retrieval loops are the points at which these components connect, and their coordination, rather than the presence of any single element, is what the model treats as consequential for student achievement. Figure 1 presents the IPRM as a conceptual design heuristic used to organise the programme redesign; it represents the design logic underpinning the reform rather than a tested causal pathway.

## Development of the architectural reform

### Context and rationale

The programme is a UK post-registration specialist nursing course delivered in a specialist ophthalmology clinical setting. It recruits registered nurses from multiple regional healthcare organisations, with comparable proportions of internally and externally sponsored participants. Demand consistently exceeds available places, with applicant-to-place ratios of approximately 2:1, reflecting workforce pressures in technically complex specialist domains.

In this high-accountability context, credible demonstration of specialist competence depends on coherence across learning design, assessment architecture, and clinical verification processes as much as on curriculum content. The curriculum comprises two integrated modules: a theory module addressing core specialist principles and a clinical practice module focused on supervised competency development. Clinical outcomes align with nationally recognised specialist competency frameworks and practitioner standards, notably the Ophthalmic Common Clinical Competency Framework developed by the Royal College of Ophthalmologists [24], positioning the programme within established regulatory expectations.

Across six pre-redesign cohorts (2018–2021), achievement distributions remained stable, with outcomes clustering near the pass-merit boundary. While overall pass rates were consistently high, the limited distribution of higher attainment classifications raised questions regarding whether features of programme architecture constrained the demonstration of higher-level achievement.

### Pre-redesign structure and identified challenges

By 2021, review of cohort-level achievement patterns and student feedback prompted examination of how the assessment architecture shaped the demonstration and recognition of competence. This shifted attention from individual learner performance toward structural features of programme design.

The summative assessment model consisted of a single 4,000-word reflective or analytical essay accounting for 100% of the academic award. Although programme outcomes emphasised applied clinical reasoning, evidence synthesis, and professional communication, graded evaluation was mediated almost exclusively through extended written discourse.

In addition to the summative essay, students completed an extensive portfolio including competency documentation, reflective accounts, workbook activities, placement reflections, and a work-based presentation. Mandatory written components totalled approximately 19,000 words, the majority of which carried formative rather than summative weighting. However, these elements were not configured as a coordinated evidentiary system; formative and summative components operated in parallel, and clinical competency verification remained structurally independent of academic grading decisions.

The pre-redesign configuration was characterised by (i) concentration of summative weighting in a single written modality, (ii) high cumulative writing volume without proportional summative contribution, (iii) limited alignment between the assessment format and the communicative and applied demands of specialist practice, and (iv) separation of academic grading and clinical competency verification without coordinated integration. Together, these structural features prompted consideration of whether greater architectural coherence could support a more authentic and proportionate demonstration of advanced capability: the question this redesign was intended to address.

### Guiding principles for redesign

The reform was guided by established educational perspectives enacted as integrated design principles rather than isolated interventions. Constructive alignment informed the recalibration of assessment formats to better reflect intended professional outcomes, particularly applied reasoning and professional communication [7]. Programmatic assessment reframed evaluation as a coordinated architecture rather than a collection of disconnected tasks, emphasising a clearer distinction between formative and summative purposes and proportional decision-making across evidence sources [8, 22].

Cognitive load theory foregrounded the consequences of high-volume, fragmented assessment structures, prioritising consolidation and depth over quantity [13]. Competency-based education emphasised staged demonstration of integrated capabilities and supported diversification of assessment modalities, including oral and case-based formats aligned with authentic specialist practice [9, 10, 12]. These principles were enacted concurrently across programme domains, with the intention of achieving structural reconfiguration rather than incremental, component-level adjustment.

### Coordinated reconfiguration of programme architecture

From early 2022, the programme underwent coordinated reconfiguration across four interdependent domains: assessment architecture, learning and feedback design, clinical organisation, and workload sequencing. Changes were embedded within formal programme artefacts, including the module handbook, competency framework, and a structured preparatory workbook. The reform was implemented as a programme-level package and refined across three subsequent cohorts. 

#### Assessment architecture

Summative assessment was restructured into two equally weighted components. Within the theory module (50%), the 4,000-word essay was replaced by a 20-minute recorded presentation (up to 40 slides) followed by a 5-minute viva. This format was designed to elicit evidence synthesis, applied clinical reasoning, and professional communication under questioning.

Within the practice module (50%), multiple short reflective tasks were consolidated into a single 3,000-word structured clinical case analysis. Using an established reflective framework of the student’s choosing (for example, Gibbs’ Reflective Cycle [25] or Johns’ Model of Structured Reflection [26]), this assessment required integration of evidence to support clinically meaningful reasoning processes rather than discrete topic coverage, thereby strengthening coherence within applied specialist practice. 

#### Embedded retrieval and formative feedback architecture

A structured preparatory workbook was introduced before each teaching session to activate prior knowledge and support cognitive readiness. Teaching adopted a flipped-learning approach, with face-to-face sessions focused on clinical application, case discussion, and problem-solving rather than content transmission [27].

Low-stakes multiple-choice quizzes were embedded at the start of each teaching day to support retrieval practice and identify conceptual gaps. Individualised formative feedback was provided for all quizzes, and students had access to scheduled one-to-one tutorials for targeted support. Formative activities were explicitly positioned as learning scaffolds rather than parallel grading mechanisms. 

#### Clinical organisation and competency processes

Clinical learning was reorganised through a protected six-week supernumerary placement incorporating structured rotation across core service areas. Competency assessment was aligned to a five-level developmental framework reflecting progressive independence.

Clinical competency verification remained mandatory for progression, but was formally separated from academic grading to clarify purpose and decision-making processes. Although grading and clinical sign-off remained distinct to preserve regulatory integrity, both were mapped to the same developmental framework, reducing divergence in evidentiary standards across domains. 

#### Workload sequencing and programme timeline

Formative activities were retained as learning scaffolding but did not contribute to summative grading. The programme duration was extended from 18 to 20 weeks to support sequencing, supervision, and access to tutorial support.

Collectively, these changes represent coordinated architectural reform spanning assessment weighting, modality distribution, competency verification, workload allocation, and retrieval-based sequencing. Rather than adjusting isolated components, the redesign reconfigured the programme as an integrated system intended to support coherence between intended specialist capabilities and the mechanisms through which competence was demonstrated and recognised.

Table 1 summarises how identified structural tensions were addressed through coordinated, theory-informed architectural action.

**Table 1:** Architectural reform: challenges and redesign across programme phases

Theoretical Principle	Structural Challenge Identified	Pre-Redesign Architecture (2018–2021; 6 cohorts)	Coordinated Architectural Redesign (2022–2023; 3 cohorts)
Constructive alignment	Misalignment between specialist competencies and assessment modality	Single 4,000-word essay (100% weighting); applied reasoning and professional communication assessed primarily through extended written discourse	Two-component summative structure (50/50): 20-minute recorded presentation with five-minute viva to elicit applied reasoning and professional communication; 3,000-word structured clinical case analysis aligned to practice-based reasoning
Programmatic assessment	Fragmented assessment system and parallel academic/clinical processes	Academic grading and clinical competency sign-off operating independently; ~19,000 cumulative words across formative and summative tasks; limited integration of evidence sources	Clear separation of academic grading and clinical verification with aligned evidentiary framework; consolidation of tasks; formative activities repositioned as learning scaffolds; structured six-week supernumerary placement supporting staged competency progression
Cognitive load theory	High assessment volume and concentrated summative weighting	Multiple written tasks (~ 15,000 formative words plus 4,000-word summative essay); overlapping deadlines; single high-stakes summative assessment	Consolidation of reflective tasks; reduced cumulative written volume; redistributed summative weighting; embedded retrieval quizzes with individualised feedback; programme duration extended (18 to 20 weeks) to support sequencing
Competency-based education	Limited modality diversity and binary representation of clinical competence	Predominantly written summative modality; clinical competence verified through pass/fail sign-off without explicit developmental staging	Diversified assessment modalities (oral presentation + viva and structured case analysis); five-level developmental competency framework reflecting progressive independence; assessment mapped to staged capability development
Assessment for learning / feedback integration	Limited structured feedback loops and weak integration of formative activity	Feedback primarily linked to final summative submission; extensive formative portfolio elements loosely integrated	Low-stakes retrieval quizzes at each teaching session; individualised formative feedback; scheduled one-to-one tutorials; structured preparatory workbook; formative tasks explicitly aligned to outcome clusters and competency framework

## Methods

### Study design

A retrospective observational cohort comparison examined differences in educational outcomes before and after programme-level structural redesign. Nine consecutive cohorts were included: six completing the traditional programme (2018–2021; *n* = 73) and three completing the redesigned programme (2022–2023; *n* = 33). Reporting was informed by the Strengthening the Reporting of Observational Studies in Epidemiology (STROBE) statement [28].

Analyses were restricted to first-attempt summative assessment outcomes to ensure comparability across cohorts and avoid potential bias introduced by resubmissions. Data relating to student experience, perceived workload, or satisfaction were not systematically collected and are therefore not included in this analysis.

### Setting and participants

Participants were registered nurses employed within specialist ophthalmic services across multiple National Health Service (NHS) Trusts in England and Wales. Cohort documentation from both programme phases indicated consistent representation from tertiary eye hospitals and regional ophthalmic departments within a stable inter-organisational referral network.

All participants were practising registered nurses with approximately 2–10 years of post-registration clinical experience. Entry requirements, employer sponsorship processes, and clinical role expectations remained unchanged across cohorts. Admission procedures, including verification of clinical scope and managerial endorsement, were maintained throughout the study period. Completion rates were comparable across phases (approximately 90%), suggesting similar levels of retention and progression across programme models.

### Assessment procedures

Summative assessment across both programme architectures was governed by structured institutional marking descriptors aligned with Level 7 academic standards (Level 7 of the UK Framework for Higher Education Qualifications corresponds to master’s-level study) [29]. Assessment domains included: (1) application of knowledge and understanding; (2) cognitive skills; (3) professional and practical skills; and (4) transferable skills.

All summative assessments were independently double-marked. Discrepancies exceeding 5% points required formal reconciliation. External moderation and institutional quality assurance procedures remained consistent throughout the study period. Marking criteria and grade band descriptors remained unchanged across cohorts, aside from the revised pass threshold associated with the qualification-level transition.

Grade classifications followed institutional conventions: Pass (50–59%), Merit (60–69%), Distinction (70–85%), and Starred Distinction (≥ 86%). All reported outcomes reflect first-attempt performance only. The core marking team and moderation structures remained stable across programme phases.

### Data analysis

During the study period, the programme transitioned from undergraduate-level provision to master’s-level provision, accompanied by an increase in the institutional pass threshold from 40% to 50%. To ensure analytical comparability, all outcomes were recalculated using a standardised 50% pass threshold across both programme phases. This standardisation supports like-for-like comparison of score distributions but is an analytic convention rather than a reconstruction of contemporaneous academic rules: pre-redesign students studied and were assessed under a 40% pass threshold, and rates reported against the 50% threshold for those cohorts do not represent the institutional pass or failure rates in force at the time.

Primary outcomes included mean and median overall achievement, grade distribution, distinction rate (≥ 70%), and the proportion of scores below the standardised 50% pass threshold. The unit of analysis was the individual student’s first-attempt overall summative score.

Between-group differences in mean scores were examined using Welch’s t-test to account for unequal group sizes and potential variance heterogeneity. Distributional assumptions were assessed using Shapiro-Wilk tests, and Mann-Whitney U tests were conducted as non-parametric sensitivity analyses. Effect sizes were calculated using Cohen’s d adjusted for unequal group sizes.

Categorical outcomes, including distinction rates and rates below the standardised threshold, were analysed using Fisher’s exact test because of small cell counts in the redesigned group. Absolute differences in proportions are reported. No demographic variables or prior academic performance data were available for covariate adjustment.

Between-group inference rests primarily on the continuous mark distributions; threshold-based classifications are reported descriptively, and classification against the 40% institutional threshold in force for the earlier cohorts is not reported. All statistical tests were two-tailed with α = 0.05. No interim analyses were conducted. Analyses were performed using R (version 4.5.2) [30]. Given the retrospective design and the multi-component nature of the redesign, this evaluation was not designed to isolate the effects of individual reform components; the analysis therefore treats the redesign as a historically situated programme-level transition rather than as a controlled intervention.

### Ethical considerations

This study analysed routinely collected programme-level academic outcome data that were fully anonymised prior to analysis. No additional data were collected, and no participant contact occurred. The study used data generated through routine programme delivery and review, and was conducted within established institutional governance frameworks.

In accordance with UK Health Research Authority guidance, formal research ethics committee approval was not required because the analysis used fully anonymised routine educational data and did not involve participant contact or the collection of identifiable personal data. Student confidentiality was protected throughout: outcome records were aggregated at cohort level and stripped of identifiers before analysis, and no individual student is identifiable in the dataset or in any reported result.

### Researcher positionality

The author had professional involvement in the programme during the period under study and contributed to the structural redesign evaluated here. This relationship is reported explicitly because it may shape the framing and interpretation of the findings. The outcomes analysed were routinely collected programme data, anonymised and aggregated at cohort level before analysis. Summative marks were generated through established institutional procedures (independent double marking, reconciliation where required, and external moderation), which were separate from the present analysis and remained stable across the study period. The analytic approach, including the standardised 50% pass threshold and the pre-specified outcomes, was fixed before group comparisons were examined. These safeguards mitigate, but cannot remove, the interpretive risks associated with insider evaluation, not least because the author contributed to shaping the assessment architecture whose outcomes are analysed here. The findings are therefore presented as a transparent retrospective programme evaluation rather than an independent external appraisal, and are interpreted with this in mind throughout the Discussion.

## Results

Across nine consecutive cohorts (*n* = 106), academic achievement differed substantially following implementation of the redesigned programme architecture. Under the traditional structure (*n* = 73), mean achievement was 57.4% (SD = 10.6; median 56.0%). Distinction-level performance (≥ 70%) was achieved by 15.1% of students (11/73), while 19.2% (14/73) scored below the standardised 50% pass threshold, a rate defined against the analytic threshold rather than the 40% institutional pass mark under which these cohorts were assessed.

Under the redesigned structure (*n* = 33), mean achievement increased to 71.1% (SD = 8.1; median 71.9%). Distinction-level performance rose to 60.6% (20/33), and no students scored below the 50% threshold.

The between-group mean difference was 13.7% points (95% CI 10.0–17.4), which was statistically significant (Welch’s t-test, *p* < 0.001) with a large effect size (Cohen’s d = 1.39). The Mann-Whitney U test confirmed this difference (*p* < 0.001). Distinction rates differed by 45.5% points (Fisher’s exact test, *p* < 0.001), and rates below the standardised threshold by 19.2% points (*p* = 0.005).

Observed score distributions were higher across the performance spectrum under the redesigned architecture, with the interquartile range (IQR) increasing from 52.0 to 65.0% to 65.0–77.0%; differences were observed across achievement levels rather than being restricted to high-performing students. Variability was lower (SD 8.1 vs. 10.6), consistent with greater convergence in achievement alongside the higher mean.

**Fig. 2:**
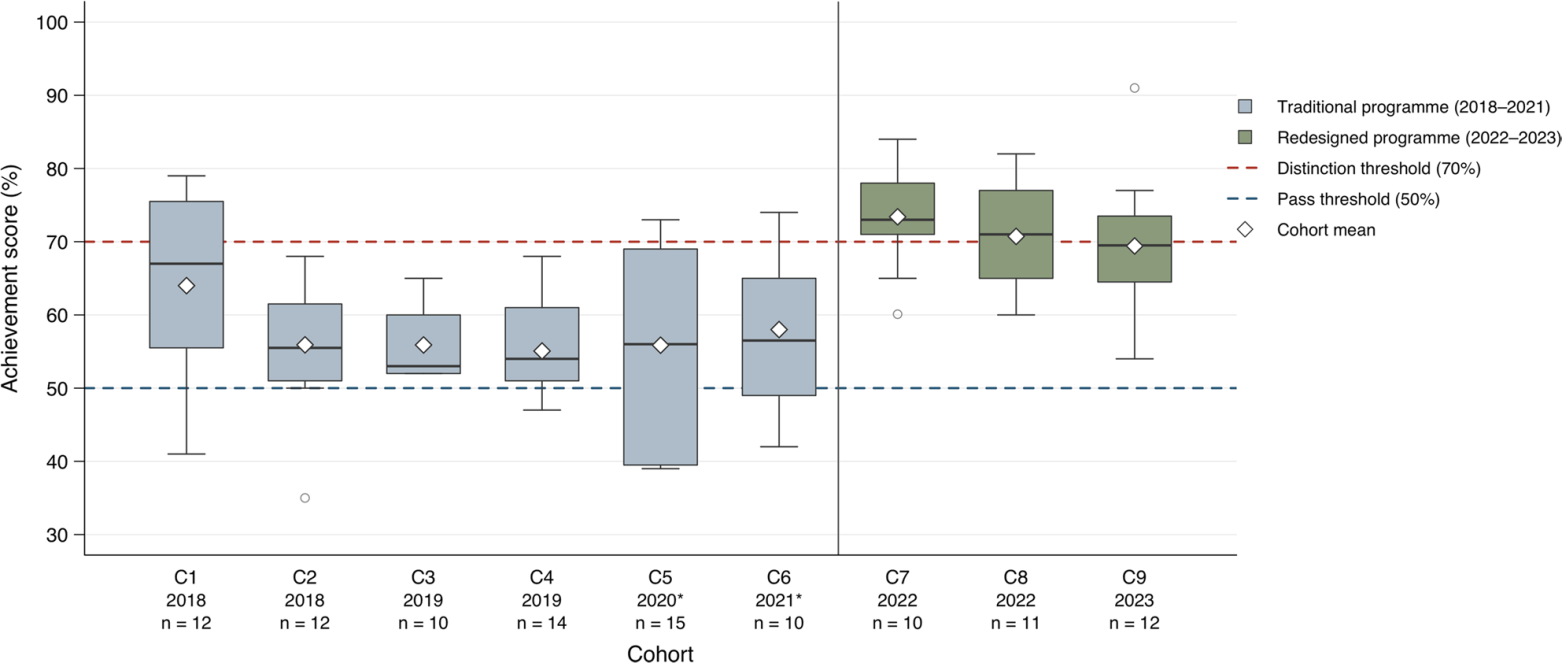
Student achievement by cohort, 2018–2023. Boxes represent the interquartile range; horizontal lines indicate the median; whiskers extend to 1.5 × IQR; open circles denote outliers; diamonds indicate cohort means. Dashed lines indicate the analytically standardised pass threshold (50%) and the distinction threshold (70%) (see Sect. "Data analysis"). The vertical line separates the traditional programme (C1–C6, *n* = 73) from the redesigned programme (C7–C9, *n* = 33). Cohorts C5–C6 were delivered during the COVID-19 pandemic (2020–2021)

Achievement patterns remained stable across the six traditional cohorts and were followed by consistently higher performance across all three redesigned cohorts (Fig. 2), including those delivered outside pandemic disruption. Table 2 summarises the between-group comparisons in overall achievement and grade outcomes. Taken together, these descriptive results, presented in Fig. 2; Table 2, indicate that the observed differences were distributed across the achievement spectrum rather than concentrated among high-performing students.

**Table 2:** Student achievement outcomes by programme model

Outcome	Traditional (*n* = 73)	Redesigned (*n* = 33)	Between-group comparison
Mean (SD)	57.4% (10.6)	71.1% (8.1)	Mean difference 13.7% points (95% CI 10.0–17.4); Cohen’s d = 1.39
Distinction ≥ 70%	11 (15.1%)	20 (60.6%)	Difference 45.5% points; *p* < 0.001
Below standardised pass threshold (< 50%)	14 (19.2%)	0 (0%)	Difference 19.2% points; *p* = 0.005

Between-group comparisons used Welch’s t-test for mean scores and Fisher’s exact test for categorical outcomes. Mann-Whitney U confirmed differences in mean scores. A standardised 50% pass threshold was applied to both periods; rates below 50% for the traditional cohorts are defined against this analytic threshold and do not represent institutional failure rates under the 40% pass mark then in force

## Discussion

This study evaluated the outcomes of a coordinated programme-level redesign aimed at reducing structural fragmentation and strengthening alignment across curriculum components. In doing so, it addresses a broader question in health professions education: whether coordinated programme architecture is associated with sustained and distribution-wide shifts in educational outcomes. Sustained higher learner achievement was observed across successive cohorts in the period following implementation of the redesigned structure. These findings are theoretically coherent with principles derived from constructive alignment [7], programmatic assessment [8], and competency-based education [9, 10]. However, given the observational cohort-comparison design, the results should be interpreted as evidence of association rather than causation. Contextual, institutional, and societal factors may also have contributed to the observed trends.

### Interpreting structural reform in context

Although the dataset is historical, it captures a complete sequence of nine cohorts before and after a defined programme-level redesign. Its value therefore lies less in describing current programme performance than in examining whether achievement patterns changed around a documented structural reform. The time elapsed since the most recent cohort nonetheless limits claims about current applicability. The pattern and consistency of the observed differences suggest that programme architecture may be associated with learner performance at a programme level, beyond what might be expected from isolated pedagogical adjustments. This supports the argument that educational outcomes may be influenced as much by how programmes organise assessment, progression, and workload over time as by teaching methods or curriculum content. The literature on curriculum drift indicates that programmes evolving through incremental modification can accumulate misalignment between outcomes, assessment demands, and workload expectations, thereby reducing coherence over time [2, 3, 17, 31]. In contrast, structural redesign efforts that intentionally re-map competencies, recalibrate assessment weighting, and sequence learning progressively have been associated with clearer developmental trajectories and improved alignment [4, 19].

The redesign evaluated in this study targeted several identified sources of fragmentation, including concentrated summative weighting, high cumulative written workload, limited modality diversity, and parallel academic and clinical processes. Within competency-based frameworks, coherent integration of outcomes and assessment is central to ensuring that progression reflects demonstrated capability rather than credit accumulation alone [9, 12]. The redesign operationalised these principles, organised through the IPRM, by aligning curriculum sequencing, assessment architecture, and developmental staging across the programme lifecycle.

From a programmatic assessment perspective, redistributing assessment encounters and strengthening longitudinal decision-making structures may reduce reliance on single high-stakes tasks while enhancing coherence and defensibility of competency judgements [8, 22]. Although reliability metrics were not directly examined in this study, the move towards an integrated assessment architecture aligns with established principles of triangulated, feedback-informed evaluation systems.

Cognitive load theory provides an additional explanatory lens [13]. In post-registration contexts, poorly synchronised deadlines and concentrated written tasks may amplify extraneous cognitive load, encouraging surface approaches to learning [5, 6]. Redesign approaches that redistribute workload and streamline assessment demands have been associated with improved engagement and performance in integrated curricula [14, 15]. The sustained upward shift in achievement observed across cohorts is therefore consistent with mechanisms intended to reduce structural overload and clarify progression expectations.

### Alternative explanations

Alternative explanations must be considered. Improvements in achievement may reflect shifts in marking practices or calibration over time rather than substantive gains in capability. The programmatic assessment literature highlights the importance of governance and calibration in maintaining consistency of evaluative judgement [22]. Although marking descriptors and moderation procedures remained stable, subtle cultural shifts cannot be excluded.

The change in assessment modality constitutes a further source of confounding through measurement non-equivalence. The pre-redesign award rested entirely on a single extended essay, whereas the redesigned award combined an oral presentation with viva questioning and a structured case analysis; the two phases therefore measured achievement through different evidentiary bases. Oral and viva-based formats may offer a different opportunity to demonstrate applied reasoning and professional communication than extended written discourse, and markers may respond differently to these formats even when formal marking criteria remain stable [7]. The redesign may thus have improved alignment between assessment and intended outcomes while simultaneously limiting comparability across phases: the observed difference cannot be attributed to structural redesign independently of the assessment formats through which achievement was measured.

Pandemic-related disruption represents an additional contextual factor. Widespread adaptation of assessment formats during COVID-19 has been documented across health professions education [32]. Although sensitivity analyses suggested that pandemic-period cohorts did not fully account for the observed differences, residual contextual effects cannot be ruled out.

The qualification-level transition itself constitutes a further alternative explanation. Between the two phases, the programme moved from undergraduate-level to master’s-level (Level 7) provision, and this repositioning coincided with the structural redesign. Beyond the change in pass threshold, the transition may have altered student expectations and preparation, the academic standing of the award and therefore applicant motivation, and the calibration of markers against level descriptors. Because these changes occurred together, the contribution of the qualification-level transition cannot be separated analytically from that of the architectural reform.

Finally, cohort variation, including differences in prior academic preparation, entry characteristics, or workplace pressures, may independently influence performance trends [33, 34]. The present design does not permit adjustment for these variables. Taken together, these considerations reinforce the case for cautious interpretation.

### Implications for practice

Notwithstanding these uncertainties, the findings contribute to emerging evidence that multi-component, system-level redesign may support coherence within post-registration nursing education and may offer transferable design principles for other health professions programmes facing similar fragmentation [17, 31]. For programme leaders, the results suggest that addressing fragmentation may require coordinated reform across assessment weighting, sequencing, workload distribution, and competency staging rather than isolated pedagogical innovation.

Rather than evidence that a particular model produced higher performance, the study offers a historically situated analysis of a complex curriculum transition. Its contribution is to make visible the architectural decisions, assessment trade-offs, and support requirements that accompany the movement from locally developed specialist training to academically accredited master’s-level provision.

Three lessons may be relevant to similar post-registration specialist programmes. First, transition to master’s-level provision asks more of a programme than academic accreditation alone: the clinical education workforce that supports learners in practice needs preparation commensurate with the new academic demands. Second, consolidating fragmented assessment may strengthen coherence, but a change in assessment modality also changes how achievement is demonstrated, and apparent gains must be read with the caution set out above. Third, alignment with a national competency framework can strengthen the professional legitimacy of specialist practice, yet it requires explicit attention to nursing identity, clinical autonomy, and the distinction between academic achievement and practice capability, a distinction this programme preserved by keeping academic grading and clinical sign-off separate.

The IPRM provides a structured heuristic to support such coordination and is intended as a transferable programme-design lens rather than a nursing-specific prescription. By integrating constructive alignment, programmatic assessment principles, competency-based progression, and workload calibration within a unified framework, it offers a practical approach to architectural redesign. Its value lies in guiding coherent decision-making across programme components rather than prescribing uniform solutions. The present study cannot establish whether the IPRM, as distinct from the wider redesign package, contributed to the observed outcome pattern; the model should be read as a way of organising reform activity rather than as evidence of causal effectiveness. In specialist post-registration education, where learners must integrate advanced clinical judgement with academic demands while remaining in demanding clinical roles, attention to structural coherence is particularly relevant for supporting sustainable progression.

### Limitations

This study has several limitations, which fall into four broad categories. First, and most importantly, the evaluation was conducted by an author who was professionally involved in the programme and contributed to the reform under study (Sect.  "Researcher positionality"). Although the analysis relied on anonymised, routinely collected outcomes and on double-marking, reconciliation, and external-moderation procedures that were independent of the author and unchanged across the period, this involvement may have disposed the interpretation towards a favourable reading, and the findings are best understood as a transparent retrospective programme evaluation rather than an independent external appraisal.

Second, the design is retrospective and observational. There was no randomisation, no external control group, and no adjustment for demographic characteristics or prior academic performance, as these data were not available; in addition, analyses were restricted to programme completers (approximately 90% of enrolees in both phases), so attrition-related selection effects cannot be excluded. The single-institution UK setting, the small number of students in the redesigned group (*n* = 33 across three cohorts), and the change in assessment modality between phases mean the two periods were not evaluated through identical instruments. Causal inference is therefore not possible.

Third, the dataset is historical. The most recent cohort outcomes pre-date this report by several years, so the findings describe a defined past period rather than current programme performance. During the period studied, the programme also transitioned from undergraduate to master’s-level provision with a corresponding rise in the institutional pass threshold from 40% to 50%; outcomes were recalculated using a standardised 50% threshold across both phases to support comparability, but this standardisation is an analytic convention rather than a reconstruction of the academic rules under which the earlier cohorts studied, and failure rates reported for those cohorts against the 50% threshold do not represent the institutional failure rates in force at the time. The qualification-level transition is therefore a substantive confounder rather than a statistical technicality and, together with pandemic-period delivery and any unmeasured changes in faculty, regulation, or marking culture, may have contributed to performance differences independently of structural redesign.

Fourth, the outcomes captured are academic. Summative marks do not directly measure clinical competence, workplace performance, patient outcomes, or long-term professional impact, and no systematic data on student experience, perceived workload, or perceived fairness of assessment were collected, and no direct measure of cognitive load was obtained; cognitive load therefore remains a theoretical rationale rather than an empirically tested mechanism in this study. Accordingly, the findings should not be interpreted as evidence of improved clinical competence, patient care, service quality, or workforce outcomes.

### Future research

Further research should examine longitudinal achievement alongside workplace-based performance indicators and programmatic assessment data [35, 36]. Linking programme-level redesign to clinical performance, service indicators, patient outcomes, and cost-effectiveness would address the persistent gap between educational reform and demonstrable practice impact [16]. Studies exploring which specific redesign components exert the strongest influence on coherence and progression would strengthen implementation guidance. Multi-site evaluations of IPRM implementation across differing institutional and regulatory contexts are needed to assess generalisability. Qualitative exploration of student and educator experiences may further clarify perceived workload, clarity of progression, and evaluative development [37]. Quasi-experimental or prospective designs would help strengthen causal inference.

## Conclusion

This single-programme retrospective cohort study observed higher first-attempt academic achievement following a complex redesign of a United Kingdom post-registration specialist ophthalmic nursing programme. Because the redesign coincided with changes in qualification level, assessment modality, assessment architecture, and programme delivery, the findings cannot establish causal effects of the IPRM, or of the structural redesign, independently of these concurrent changes. The pattern nonetheless suggests that programme-level architecture (how outcomes, assessment, competency verification, and workload are organised in relation to one another) deserves attention alongside curriculum content in post-registration specialist nursing education. By integrating alignment, programmatic assessment, competency progression, and workload calibration within a unified framework, the IPRM is offered as a conceptual design heuristic for organising programme-level reform in complex clinical education contexts, not as a validated explanatory model. These conclusions are drawn from a single specialist ophthalmic nursing programme over a defined historical period and from an evaluation in which the author was professionally involved; prospective, independent, multi-site evaluation is required before any claim of effectiveness or transferability can be made.

## Data Availability

The datasets analysed during the current study are not publicly available because they contain institutionally governed educational data and are subject to confidentiality restrictions. De-identified aggregate data may be available from the corresponding author on reasonable request and subject to institutional approval.
